# Feasibility and reproducibility of handheld and table-mounted optical coherence tomography in children with craniosynostosis

**DOI:** 10.1038/s41433-026-04317-3

**Published:** 2026-02-24

**Authors:** Sohaib R. Rufai, Dermot Roche, Riddhi Shenoy, Oliver R. Marmoy, Vasiliki Panteli, Dorothy A. Thomson, Robert H. Henderson, Irene Gottlob, Mervyn G. Thomas, Catey Bunce, Noor ul Owase Jeelani, Richard Bowman, Frank A. Proudlock

**Affiliations:** 1 Clinical and Academic Department of Ophthalmology, Great Ormond Street Hospital for Children NHS Foundation Trust and UCL Great Ormond Street Institute of Child Health, London, UK; 2 Craniofacial Unit, Great Ormond Street Hospital for Children NHS Foundation Trust and UCL Great Ormond Street Institute of Child Health, London, UK; 3 The University of Leicester Ulverscroft Eye Unit, Leicester Royal Infirmary, Leicester, UK; 4 Epidemiology and Population Health, London School of Hygiene & Tropical Medicine, London, UK

**Keywords:** Nervous system, Biomarkers, Biomarkers, Tomography, Neuroscience

## Abstract

**Background:**

Optical coherence tomography (OCT) can be a valuable tool for non-invasively monitoring the optic nerve status in children with craniosynostosis. However, it is currently unknown whether optic nerve parameters derived from handheld OCT are comparable to those derived from table-mounted OCT, which is more widely used. This study aims to assess the feasibility and reproducibility of handheld and table-mounted OCT in craniosynostosis.

**Methods:**

This was a cross-sectional study conducted at Great Ormond Street Hospital (GOSH), London. Twenty children aged 4–18 years with a clinical/genetic diagnosis of craniosynostosis were included. Bilateral optic nerve head OCT imaging was performed using the Spectralis (Heidelberg Engineering), followed by the handheld Envisu C2300 (Leica Microsystems). Primary outcome measures were quantitative cup, disc, rim and peripapillary parameters. Intraclass correlation coefficients (ICC) and coefficient of variation (CoV) were calculated for each quantitative OCT parameter.

**Results:**

20 children (100%) were successfully recruited. Median age at the time of OCT examination was 6 years (range: 4–16; IQR: 5–8). Ten participants (50%) were female. Seven participants (35%) had syndromic craniosynostosis and 13 participants (65%) had non-syndromic craniosynostosis. Bilateral imaging success was 100% for both machines. ICCs were good-to-excellent for all parameters, ranging from 0.81 to 1.00. The coefficient of variation was low for all parameters.

**Conclusions:**

OCT imaging of the optic nerve is feasible in school-aged children with craniosynostosis and comparable between the Spectralis and handheld Envisu OCT. This could allow comparison and pooling of data between the two machines, greatly enhancing patient care and future research.

## Introduction

Craniosynostosis is characterised by premature fusion of the cranial sutures. Its prevalence is approximately 5.9 per 10,000 live births [1]. Craniosynostosis is often associated with raised intracranial pressure (ICP), known as intracranial hypertension (IH). IH can damage the brain and vision if unaddressed, hence it is of paramount importance that IH is recognised and treated promptly. Direct ICP measurement is invasive, involving admission to hospital, anaesthesia and surgical risks. Furthermore, it is difficult to obtain serial invasive ICP measurements [2].

Optical coherence tomography (OCT) can non-invasively detect papilloedema due to IH [3]. Swanson et al. [4] and Kalmar et al. [5] have reported that OCT can detect IH in children with craniosynostosis under general anaesthesia. The Envisu C2300 handheld OCT (Leica Microsystem, Wetzlar, Germany) is a child-friendly OCT machine designed for use in unsedated children in the clinic setting [6, 7]. Our group has demonstrated that handheld OCT is feasible and repeatable in unsedated children with craniosynostosis [8, 9]. Patel et al. [10] have produced a normative handheld OCT database of the optic nerve head from birth to adolescence. Only a minority of hospitals possess handheld OCT, whereas the Spectralis table-top OCT (Heidelberg Engineering, Heidelberg, Germany) is far more widely used, as evidenced by over 1500 PubMed [11] results for the search term ‘Spectralis’ as of September 1^st^, 2025.

No study has yet assessed the feasibility and reproducibility of handheld and table-mounted OCT in children with craniosynostosis, which could greatly enhance patient care and future research. This could permit pooling of data between the two machines, comparison of table-mounted OCT data against the handheld OCT normative database produced by Patel et al. [10], and comparison between handheld OCT measurements in infants with table-mounted OCT as they grow older. Furthermore, this is a valuable cohort in which to robustly assess the reproducibility of optic nerve parameters, due to a wider range of optic nerve morphology demonstrated in children with craniosynostosis [12].

The primary objective of this study is to assess the reproducibility of quantitative OCT parameters of the optic nerve between the Spectralis and Envisu C2300 machines. The secondary objective is to assess inter-machine agreement using qualitative OCT signs of IH.

## Methods

### Study design and participants

This was a cross-sectional study that took place at Great Ormond Street Hospital for Children (GOSH), London. This study adhered to the tenets of the Declaration of Helsinki. Informed consent was obtained for all participants. Ethical approval was granted by the East Midlands Nottingham 2 Research Ethics Committee (UOL0348/IRAS 105137). This study represented preliminary work supporting the Recognition of Intracranial hypertension using handheld OCT in children (RIO) Study (ISRCTN52858719) [13]. This study was reported as per the Strengthening the Reporting of Observational Studies in Epidemiology (STROBE) statement [14].

Twenty participants were approached for recruitment between October 21, 2021, and March 24, 2022. Participants were recruited from the craniofacial ophthalmology clinic (*n* = 19) or surgical admissions ward prior to ICP monitoring (*n* = 1). School-aged participants, aged 4–18 years, with a clinical/genetic diagnosis of craniosynostosis were included. Pre-school children aged under 4 years were excluded due to a low likelihood of successful OCT imaging using table-top OCT machines, which would impede our reproducibility analysis.

Considering that craniosynostosis is a rare condition [1], we determined that collecting 80 images from a cohort of 20 patients would be sufficient for this study. Attempting to recruit a larger cohort would leave insufficient time to fulfil the objectives of the RIO Study [13].

### OCT image acquisition and analysis

**Fig. 1:**
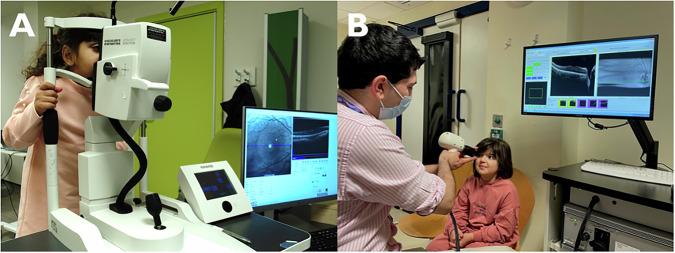
Two OCT Machines. **A** Spectralis (Heidelberg Engineering); **B** Envisu C2300 (Leica Microsystems). Consent to publish was obtained for both patients.

Two non-contact spectral-domain OCT devices were utilised in this study (Fig. 1): the Spectralis and the Envisu C2300 handheld OCT. These systems provide axial resolutions of 3.9 µm and 3.3 µm, respectively.

With respect to the Envisu C2300, scans were obtained using a 12 ×8 mm scanning window with an acquisition time of 1.9 s, supporting rapid image acquisition with minimal quality compromise to avoid measurement bias; the 3D raster scan protocol included 80 B-scans, with each B-scan line comprising 600 A-scans. The handheld OCT probe was held approximately two inches from the patient’s eye, with the investigator’s fingers and thumb stabilising the probe against the patient’s forehead and cheek, preventing inadvertent probe contact or image tilt. Image tilt was minimised as much as possible by an experienced investigator (SRR): (i) the investigator encouraged the patient to keep their head straight, and aligned the probe such that the handle was vertically aligned with the patient’s face and perpendicular to their jaw; (ii) the on-screen OCT images were subjectively assessed for tilt by checking whether Bruch’s membrane appeared level at the nasal and temporal sides of the scan window. The investigator’s arms were extended to provide the patient with adequate space and comfort. Handheld OCT imaging sessions took approximately 8–10 min per patient. The Spectralis protocol used a 9.1 × 9.1 mm scanning area with a 1.7-s acquisition time, and the 3D raster consisted of 97 B-scans, each with 768 A-scans. The Spectralis imaging sessions took approximately 6–8 min per patient, due to the convenience of the chin-rest and head-rest system and in-built fixation target.

All patients were imaged using the Spectralis first, followed by the Envisu C2300. All Spectralis table-mounted OCT imaging (*n* = 19) was performed by an experienced Vision Scientist (DR); all Envisu C2300 handheld OCT scans (*n* = 20) plus one Spectralis Flex OCT scan were performed by the lead investigator (SRR) due to logistical reasons. Each imaging session lasted up to 10 min per machine, aiming to acquire bilateral optic nerve head images of adequate quality for analysis. Adequate quality was defined as a tomogram where disc margins and the cup profile, including its deepest point, were clearly visualised. If image quality was inadequate, repeat scans were attempted during the same visit, or until the child ceased to participate. For the Spectralis, a built-in fixation target was used to bring the optic nerve head into the scanning window and minimise measurement bias. The Envisu C2300 handheld OCT does not feature a built-in fixation target; hence, visual fixation devices (such as cartoons on smartphones/tablets or toys) were used to minimise movement during handheld OCT imaging to minimise measurement bias. Our imaging protocol involved scanning the right eye, followed by the left eye. The *en face* view was used to locate the optic nerve head—this was navigated, frame by frame, until the central slice featuring the deepest optic cup was identified for analysis. SRR identified the central OCT slice for all Envisu C2300 images, while DR independently identified the central OCT slice for all Spectralis images.

Quantitative segmentation analysis was performed using ImageJ 1.4815 (National Institutes of Health, Bethesda, MD) [15], the ABSnake plugin [16] facilitated semi-automated identification of the internal limiting membrane contour, which was manually corrected as necessary. The lead investigator (SRR) carried out all quantitative segmentation analyses. Scale factors from Folgar et al. [17] were applied to scale measurements from Spectralis to Envisu: lateral scaling, 0.989; axial scaling, 0.975. Lateral distance measurements were reported in visual angles, as these remain stable from birth through adolescence [10].

**Fig. 2:**
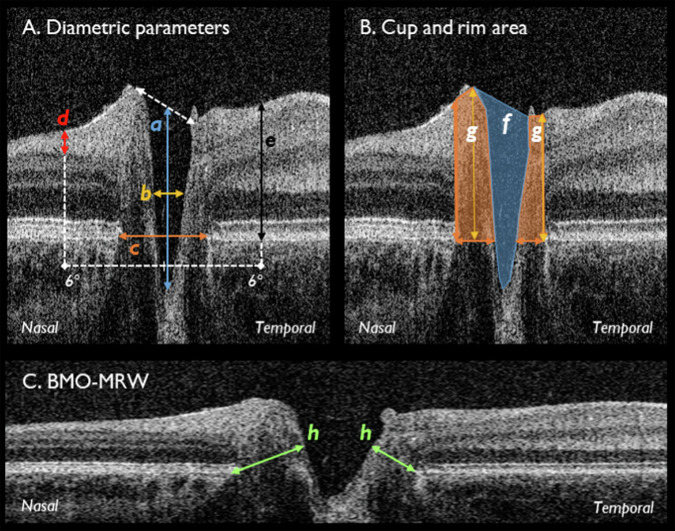
Quantitative segmentation parameters. Original images captured using the Envisu C2300 (Leica Microsystems). **A** Diametric parameters for cup, disc, RNFL and retinal thickness: a) cup depth (blue), measured from cup base to midpoint of neuroretinal peaks; b) cup diameter (amber), measured at midpoint of cup depth; c) disc diameter (orange), measured from nasal to temporal Bruch’s membrane; d) RNFL thickness (red), measured at 6° from the disc midpoint, bounded by the ILM and GCL; e) retinal thickness (black) measured at 6° from the disc midpoint, bounded by the ILM and Bruch’s membrane. **B** Cup area and rim parameters: f) cup area (blue shade), bounded by neuroretinal peaks; g) rim area (orange shade), bounded by edges of Bruch’s membrane; rim width, represented by lower-most borders of g); rim height, maximum distance between rim width and ILM represented by amber arrows; rim edges, represented by lateral borders of g). **C** Natural scale image displaying h) BMO-MRW. Nasal and temporal measurements were taken for all parameters where applicable. BMO-MRW = Bruch’s membrane opening minimum rim width; GCL = ganglion cell layer, ILM = internal limiting membrane, RNFL = retinal nerve fibre layer thickness. Source: Rufai SR, Bowman R, Bunce C, Panteli V, McLean RJ, Teli S, Gottlob I, Thomas MG, Jeelani NUO, Proudlock FA. Feasibility and Repeatability of Handheld Optical Coherence Tomography in Children With Craniosynostosis. Transl Vis Sci Technol. 2021 Jul 1;10(8):24. Open access (CC BY 4.0).

**Fig. 3:**
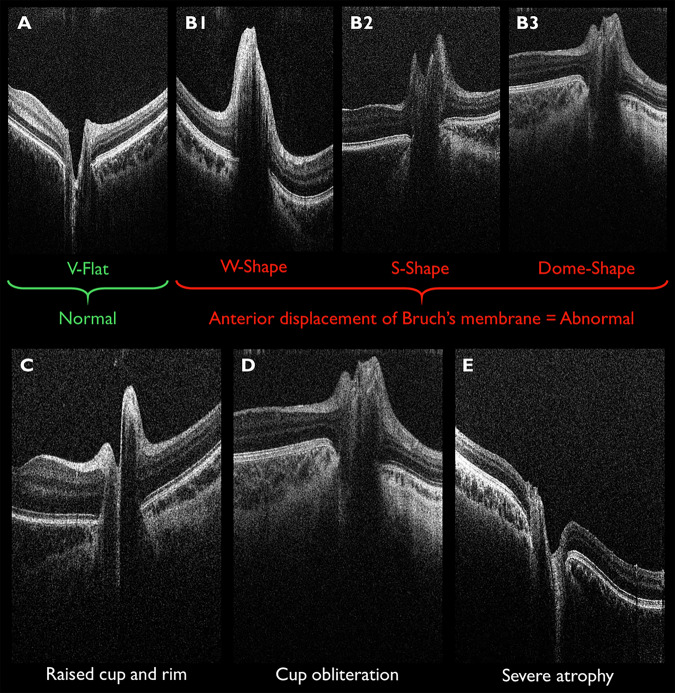
Qualitative OCT signs. The top row **A**, **B** displays possible configurations of Bruch’s membrane, graded as per criteria suggested by Sibony et al. [3]. The normal configuration of Bruch’s membrane is ‘V-flat’ (**A**), while any anterior displacement of Bruch’s membrane (**B**1-**B**3) is abnormal and suggestive of intracranial hypertension. The bottom row displays further OCT signs proposed by our group. The cup and rim are deemed raised (**C**) if the cup is above the level of the disc edges and the rim is bilaterally upward-sloping. The cup is deemed obliterated (**D**) if the criteria for raised cup and rim are met, plus the normal ‘U’ shaped profile of the cup is disturbed. Severe atrophy (**E**) is deemed present if the retinal nerve fibre layer appears extremely thin or absent.

Figure 2 displays the segmentation parameters used for quantitative segmentation analysis. Figure 3 displays qualitative OCT signs, graded by an experienced grader (SRR) and a novice grader (RS) before and after brief training. The following criteria were graded as yes/no: anterior displacement of Bruch’s membrane, raised cup and rim, cup obliteration, and severe optic atrophy. Before training, the novice grader was simply provided with Fig. 3 to grade the images. Brief training involved the experienced grader discussing the grading process with the novice grader in greater detail. All images were masked and randomised prior to grading using Excel (Microsoft Office LTSC Professional Plus 2021, Microsoft, Washington, US), to avoid investigator bias. Both Fig. 2 and Fig. 3 were created a priori, using handheld OCT images of children with craniosynostosis, collected as part of our previous and ongoing works [8, 12, 13].

### Statistical analysis

Statistical analysis was performed using SPSS Statistics 22.0. (IBM Corp., Armonk, NY). Demographic data for diagnoses, sex, age and logMAR visual acuity were summarised using descriptive statistics. Recruitment success and bilateral OCT imaging success rates were reported as percentages.

Primary outcome measures were quantitative measurements for optic nerve head scans across the two machines. Secondary outcome measures were qualitative grades for optic nerve head scans across the two machines.

With respect to the quantitative comparison, overall mean figures for the included OCT parameters were calculated and determined to be approximately normally distributed. Mean differences for the included OCT parameters between the two machines were calculated. Intraclass correlation coefficients were calculated for all OCT parameters using average measures in a two-way mixed-effects model [18]. In addition, 95% limits of agreement were calculated as mean difference ± 1.96 standard deviation of the difference. T-tests were performed to assess for systemic bias. Coefficients of variation, defined as the ratio of the standard deviation to the mean, were calculated for all OCT parameters between the two machines. An available case analysis was conducted for OCT outcome measures, as only a small number of peripapillary OCT measurements were missing.

With respect to the qualitative comparison, the images were masked and randomised prior to grading by the experienced grader (SRR) and novice grader (RS). A novice grader was employed to assess the usability and generalisability of the qualitative grading scheme (Fig. 3). Intermachine agreement per grader and intergrader agreement before and after training were reported as percentages.

## Results

### Baseline characteristics

Out of 20 children approached for recruitment, all 20 (100%) were successfully recruited. Bilateral OCT scans of the optic nerve head were successful in all children (100%) using both machines, i.e. 40 eyes of 20 patients using the Envisu C2300, and 40 eyes of the same 20 patients using the Spectralis (table-top, *n* = 19; Flex module, *n* = 1). Ten participants (50%) were male and ten (50%) were female. Median age at the time of OCT examination was 6 years (range: 4–16; IQR: 5-8). Seven patients had syndromic craniosynostosis (Crouzon, *n* = 3; Muenke, *n* = 3; and TCF12, *n* = 1). Thirteen patients had non-syndromic craniosynostosis (sagittal, *n* = 8; and multisuture, *n* = 5). Median visual acuity (logMAR) was 0.05 (range: –0.10 to 0.56; IQR: –0.02 to 0.11).

### Quantitative comparison

Reproducibility analysis was feasible in all 20 (100%) children, where both eyes were imaged using the Spectralis OCT, followed by the Envisu C2300 OCT. Reproducibility analysis (Table 1) revealed good-to-excellent reproducibility across all parameters. Only disc width, RNFL nasal thickness and RNFL temporal thickness fell below 0.90 (excellent). Percentage differences and coefficient of variation were low for all parameters.

**Table 1:** Reproducibility between Envisu C2300 and Spectralis OCT machines.

	Overall mean	Mean difference	% difference	SD of differences	95% Limits of Agreement	Bias (T-test p-value)	ICC (95% CI)	CoV
**Cup / disc parameter (***n* = **40)**								
Cup depth (µm)	337.5	-10.4	-3.1%	24.0	-57.5, 36.7	0.013	1.00 (0.99–1.00)	7.1%
Cup width (deg)	1.73	0.12	6.9%	69.4	-0.35, 0.59	0.005	0.98 (0.97–0.99)	13.9%
Cup area (µm.deg)	665	0.1	0.0%	24727	-168, 168	0.997	0.99 (0.98–1.00)	12.9%
Disc width (deg)	5.70	0.22	3.8%	109.1	-0.53, 0.96	0.001	0.81 (0.64–0.90)	6.6%
**Rim parameters (***n* = **40)**								
Nasal rim height (µm)	512.4	3.26	0.6%	36.1	–67.5, 74.0	0.572	0.98 (0.96–0.99)	7.0%
Temporal rim height (µm)	330.8	1.05	0.3%	20.5	–39.0, 41.1	0.748	0.99 (0.98–0.99)	6.2%
Rim width (deg)	5.03	0.27	5.3%	116.1	–0.52, 1.06	0.000	0.97 (0.93–0.98)	8.0%
Rim area (µm.deg)	1864	97.5	5.2%	58241	–299, 494	0.004	0.99 (0.97–0.99)	10.8%
Nasal BMO (µm)	430.3	-6.66	-1.5%	27.1	–59.7, 46.4	0.128	0.90 (0.80–0.95)	6.3%
Temporal BMO (µm)	300.7	-5.70	-1.9%	20.9	–46.6, 35.2	0.092	0.97 (0.94–0.98)	6.9%
**Peripapillary parameters (***n* = **39 for temporal,** *n* = **36 for nasal)**								
Nasal retinal thickness (µm)	271.1	–5.34	–2.0%	9.19	–23.4, 12.7	0.754	0.87 (0.75–0.93)	3.4%
Temporal retinal thickness (µm)	287.8	–7.30	–2.5%	7.06	–21.1, 6.5	0.000	0.82 (0.66–0.91)	2.5%
Nasal RNFL thickness (µm)	63.8	–0.89	–1.4%	11.7	–23.8, 22.0	0.000	0.98 (0.97–0.99)	18.3%
Temporal RNFL thickness (µm)	52.4	–2.67	–5.1%	8.35	–19.0, 13.7	0.053	0.98 (0.96–0.99)	15.9%

Key: 80 images from 20 children were included. Overall means were derived from the Envisu C2300 handheld OCT. The mean difference in OCT parameters was derived from the Envisu C2300 image versus Spectralis image, where both images were taken in the same eye during the same visit. The ICCs were obtained using a two-way mixed effects model.

*CI* confidence interval, *CoV* coefficient of variation, *BMO* Bruch’s membrane opening, *µm* micrometres, *ICC* intraclass correlation coefficient, *SD* standard deviation, *°* degrees.

### Qualitative comparison

Expert grading delivered 100% intermachine agreement (40/40). Untrained novice grading delivered 96% intergrader agreement (77/80) and 93% intermachine agreement (37/40); following training and consensus discussion, 100% intergrader (80/80) and intermachine agreement (40/40) was achieved, whereby the novice grading matched the expert grading. Of the 20 participants, one demonstrated bilateral anterior displacement of Bruch’s membrane (S-shape), seven displayed bilateral raised rim and cup, two displayed unilateral cup obliteration; all other OCTs were graded as normal, with none displaying severe optic atrophy.

## Discussion

### Main findings

To the best of our knowledge, this represents the first study comparing two OCT machines in children with craniosynostosis. This is confirmed by our systematic review [19] and more recent PubMed search on September 1st, 2025, where the search terms ‘handheld optical coherence tomography craniosynostosis’ returned no similar studies. All twenty children agreed to participate. All OCT examinations were successful, but a small amount of data was missing for peripapillary parameters. Quantitative analysis demonstrated good-to-excellent reproducibility across all OCT parameters. Qualitative analysis demonstrated excellent intermachine agreement.

### Research in context

In our study, disc width, nasal and temporal RNFL thickness demonstrated good reproducibility, with ICCs of 0.81, 0.87 and 0.82, respectively. All other OCT parameters demonstrated excellent reproducibility with ICCs greater than 0.90, in many cases approaching 1.00. The disc edges can sometimes appear less ‘sharp’, possibly leading to a degree of variation in manual segmentation. However, the coefficient of variation for disc width was low (6.6%), suggesting low variability between measurements, which were tightly clustered together.

Despite good-to-excellent reproducibility across all measures, a small degree of systematic bias was demonstrated for the following axial measures: cup depth, retinal thickness and nasal RNFL 1.4-3.1% smaller on the Spectralis. ‘Acutance’ is the measure of sharpness and thus perceptibility of the edge of an image. The cup base may have been slightly easier to visualise and pinpoint on the Spectralis due to higher acutance, with a slight overmeasurement on the Envisu, where the cup base appeared slightly ‘fluffy’, therefore harder to pinpoint. Furthermore, a higher signal-to-noise ratio may have resulted in slightly larger measures for RNFL and retinal thickness. By contrast, the cup width, disc width, rim width and rim area were 3.5–8% greater on the Envisu. This may be due to slightly higher acutance of the disc edges and cup walls using the Spectralis, resulting in slightly smaller measurements. Nonetheless, percentage differences and coefficient of variation were low for all measures and, whilst statistically significant, these figures are unlikely to be clinically significant.

Our previous study [8] included intra-machine repeatability analysis in 40 eyes of 20 children with craniosynostosis, where the same eye was imaged twice using the same Envisu C2300 machine. The current intermachine reproducibility study (Envisu C2300 versus Spectralis) has demonstrated generally similar or higher ICCs than the intra-machine (Envisu C2300) repeatability study [8]. The cup parameters all demonstrated higher ICCs in the intermachine reproducibility study, notably cup width (0.98 vs. 0.82). The rim parameter ICCs were similar in both studies. The peripapillary parameter ICCs were higher in the intermachine reproducibility study, especially for nasal RNFL (0.87 vs. 0.81) and temporal RNFL (0.82 vs. 0.77). There are a number of potential explanations for these findings. Firstly, the Spectralis table-top machine virtually eliminates operator-induced tilt, due to the chin-rest and head-rest, whereas operator-induced tilt is possible using the Envisu C2300 handheld OCT. On the other hand, inadvertent operator-induced tilt was possible in all images in the intra-machine handheld OCT repeatability study. Secondly, the lead author (SRR) has acquired increased experience in handheld OCT image acquisition and segmentation analysis in this study compared to the previous study, and the Spectralis imager (DR) is a vision scientist with 14 years’ experience in OCT imaging. Thirdly, the children were older in the inter-machine reproducibility study (mean: 6 years; range: 4–16) compared to the intra-machine repeatability study (mean: 4 years; range: 0–13); better compliance may have led to reduced image tilt and movement artefact in the older cohort. Finally, some differences may have occurred due to chance. With respect to disc width, the ICC was lower (0.82) in the inter-machine reproducibility study compared to the intra-machine repeatability study (0.91), which may have been due to variation in segmentation of the disc edges owing to higher acutance on Spectralis images, or may have occurred due to chance; in any case, the ICC still implies good reproducibility. A degree of variation in RNFL thickness can be induced due to inadvertent tilting of the handheld OCT probe [8, 20].

Folgar et al. [17] assessed the reproducibility of lateral and axial OCT measurements of one human retina phantom (Rowe model eye, Rowe Technical Designs, Orange County, California, USA), using various OCT devices, including the Envisu C2300 (Leica Microsystems) and the Spectralis (Heidelberg Engineering). They assessed the following foveal parameters: fixed lateral width, centre foveal thickness, and parafoveal thickness 1 mm to the left and right of centre. They found excellent intergrader reproducibility. Their study optimised imaging conditions in a retina phantom, producing conversion factors to allow investigators to translate quantitative OCT data from one machine to another. Their study did not assess optic nerve parameters in a model or a human eye. Our study assessed fourteen optic nerve OCT parameters in 20 children with craniosynostosis in real-world conditions.

### Limitations

Due to time constraints following the COVID-19 pandemic, our study faced unavoidable delays and recruitment challenges. Hence, the RIO Expert Advisory Group made a pragmatic decision to analyse 80 images on 20 children for the reproducibility study, to permit timely comparison with our previous intra-machine repeatability study (*n* = 20) [8]. A larger sample size would have permitted more robust reproducibility analysis and in-depth subgroup analyses by diagnosis and other demographics. One patient was imaged using the Spectralis Flex module due to logistical reasons – whilst this is essentially the same machine as the table-top Spectralis, the Flex module may have introduced other factors affecting reproducibility, such as inadvertent operator-induced tilt. A small amount of data was missing for peripapillary parameters, as these can sometimes be ‘chopped off’ if the patient does not fix optimally; thus, a larger cohort could allow more robust statistical analyses.

## Conclusions

This study has demonstrated good reproducibility of optic nerve head parameters using two optical coherence tomography machines (Envisu C2300 and Spectralis) in children with craniosynostosis. Our findings suggest that data can be compared or pooled between these two machines for future research studies [13]. These findings also support investigators using the Spectralis machine to compare their data to the normative handheld OCT dataset by dataset by Patel et al. [10] produced using the Envisu C2300 machine, provided they use the conversion factors by Folgar et al. [17]. Finally, handheld OCT measurements taken in a young infant with craniosynostosis can be compared with Spectralis measurements in the same child when they grow older. In total, these findings could enhance patient care and future research.

## Summary

### What is known about this topic

- OCT can be a valuable tool for non-invasively monitoring the optic nerve status in children with craniosynostosis

### What this study adds

- OCT imaging of the optic nerve is feasible in children with craniosynostosis and comparable between the Spectralis and handheld Envisu OCT.
- This could allow comparison and pooling of data between the two machines, greatly enhancing patient care and future research.

## Data Availability

Data available upon reasonable request.
